# Supplementary material for: Does nicotine exposure during adolescence modify the course of schizophrenia-like symptoms? Behavioral analysis in a phencyclidine-induced mice model
Source: PLoS One. 2021 Sep 29;16(9):e0257986. doi: 10.1371/journal.pone.0257986 (PMC8480744; doi:10.1371/journal.pone.0257986)
Supplement: Attachment — Submitted filename: Dutra-Tavares et al 2021 Response to Reviewers - R1.docx [file pone.0257986.s002.docx]

**JOURNAL REQUIREMENTS:**

*1. Please ensure that your manuscript meets PLOS ONE's style requirements, including those for file naming.*

**Response:** The manuscript meets PLOS ONE's style requirements.

*2. We note that you have included the phrase “data not shown” in your manuscript. Unfortunately, this does not meet our data sharing requirements. PLOS does not permit references to inaccessible data. We require that authors provide all relevant data within the paper, Supporting Information files, or in an acceptable, public repository. Please add a citation to support this phrase or upload the data that corresponds with these findings to a stable repository (such as Figshare or Dryad) and provide and URLs, DOIs, or accession numbers that may be used to access these data. Or, if the data are not a core part of the research being presented in your study, we ask that you remove the phrase that refers to these data.*

**Response:** Body mass means and standard errors are now shown in table 1. Raw data are available as supplementary material.

*3. Please include captions for your Supporting Information files at the end of your manuscript, and update any in-text citations to match accordingly.*

**Response:** Done.

__________________________________________________________________________

**REVIEWER #1:**

We are thankful for the Reviewer's positive comments. Here, we fully considered his/her comments and criticisms. Please note that, in addition to highlighting the changes to the manuscript in the revised version, below, we underlined the text that was either included or revised, and we used the strikeout format to indicate texts that were deleted from the revised version.

*Major Considerations:*

*1. It is somewhat difficult to make a thorough conclusion of the study because the experimental design may require additional controls. For instance, in my reading of the study, it appears that mice either received nicotine or saline pumps, accompanied by phencyclidine or saline injections and only received these treatments throughout multiple behavioral tests (i.e., either phencyclidine or saline). Is it possible that an acute injection of PCP on test day could induce behavioral responses that would be important for understanding the basis of nicotine-phencyclidine interactions? If so, there appear to be several groups missing in the experimental design (e.g., nicotine and saline pumps + saline injections, given PCP on test days).*

**Response:** We chose to use an established rodent model of SCHZ that evokes behavioral as well as other characteristics of SCHZ (Winship et al., 2018. Can J Psychiatry 64:5-17. doi:10.1177/0706743718773728; Bubeníková-Valešová et al., 2008. Neurosci Biobehav Rev 32:1014–1023. doi:10.1016/j.neubiorev.2008.03.012; Tizabi, 2007. Neurotox Res 12:233–246. doi:10.1007/BF03033907). In our experimental design, mice received a phencyclidine injection every day from PN38 to PN53, which includes the period mice are submitted to the battery of behavioral tests (PN48-53). With this approach, we guaranteed that the modeling of SCHZ was effective during the whole period of testing. This was confirmed by the identification of detrimental effects of phencyclidine exposure in all SCHZ-like analyzed behaviors. The Reviewer raises the possibility that an additional analysis of an acute injection of phencyclidine on test day would bring relevant information. In his regard, even though both acute and long-term exposures to phencyclidine are used to model SCHZ, the animal model based on long-term exposure shows better constructive and phenomenological validity (Bubeníková-Valesová et al., 2008. Neurosci Biobehav Rev 32:1014-23. doi: 10.1016/j.neubiorev.2008.03.012). Even though we agree with the reviewer that the investigation of the impact of an acute injection of phencyclidine in mice with a prior history of nicotine exposure could bring new information, this methodological approach (nicotine first, followed by phencyclidine on test day) does not model the onset of tobacco smoking-schizophrenia co-morbidity described in epidemiological studies (Kotov et al., 2010. Schizophr Bull 36:173-181. doi:10.1093/schbul/sbn066), which lessens its relevance. Considering both the advantages of a subchronic model, and the low probability of reproducibility of the proposed acute model in SCHZ patients, in accordance with the three Rs principle, we understand that there is not sufficient justification for adding separate cohorts of mice (one for each behavioral test) to create additional experimental groups of acute phencyclidine exposure. The Reviewer’s comment indicates that the rationale for choosing this model needs to be more clearly stated. We added a brief but detailed rationale in the introduction of the manuscript.

**Highlighted version (Introduction, page 4, line 133)**

(…) Repeated administration of rodents with phencyclidine, a non-competitive NMDA receptor antagonist, is considered the best validated and most widely used model in SCHZ research [40,41]. It produces behavioral alterations associated with the three core (positive, negative and cognitive) symptoms of SCHZ [42]; reduces prepulse inhibition (PPI), a SCHZ endophenotype [43,44] and evokes neurochemical abnormalities associated with SCHZ such as hyperactivity of the mesolimbic dopaminergic pathway and hypoactivity of the mesocortical one [42,45]. Particularly relevant to the current study, the dysregulation of the glutamatergic system has been suggested to play a role in the progression of SCHZ from the prodromal phase to psychosis onset [46–48].

1’. *Likewise, it would seem prudent to compare the effects of mice receiving PCP injections throughout behavioral testing with relevant saline controls (e.g., nicotine and saline pumps + PCP injections, given saline on test days).*

**Response:** This is a relevant issue. The objective of this study was to investigate whether a short period of nicotine exposure during adolescence changes the course of SCHZ. To reach this objective, our experimental design needed to fulfill some conditions: 1) Nicotine exposure needed to be restricted to the adolescent period, 2) The spectrum of SCHZ-like symptoms was to be investigated after the end of nicotine exposure, 3) The impact of nicotine history was to be evaluated by comparing mice exposed to phencyclidine (PCP group) with those with a prior exposure to nicotine (PCP-NIC group). Although we agree with the Reviewer in that exposure to nicotine during the behavioral tests could bring interesting information, the extension of the period of nicotine exposure to include the days of behavioral tests would compromise the interpretation of the data. In this regard, previous data demonstrate that short- versus long-term exposures may result in very distinct behavioral and neurochemical alterations (e.g. Abreu-Villaça et al., 2017. Biochem Pharmacol 144: 1-17. doi: 10.1016/j.bcp.2017.06.113; Bombardi et al., 2021. Int J Mol Sci 22: 4775. doi: 10.3390/ijms22094775; Dean et al., 2020. Sci Rep 10: 8570. doi: 10.1038/s41598-020-65382-6). We also agree with the Reviewer that further studies aiming to deepen our understanding of the basis of nicotine-phencyclidine interactions are needed. However, it is our understanding that the investigation of concomitant phencyclidine and nicotine exposures, while related to the subject covered in our study, would answer a distinct question, and, therefore, entails a distinct study. The manuscript was modified to make it clearer how the methodological approach was designed to answer our experimental question and to point out that future studies are needed to investigate other aspects of nicotine and SCHZ interactions, particularly during adolescence.

**Highlighted version (Introduction, page 5, line 145)**

Here, we used mice models to elucidate the impact of a short course of adolescent nicotine exposure across the spectrum of SCHZ-like symptoms. Phencyclidine administered on a daily basis guaranteed the development of deficits associated with SCHZ. As for nicotine, exposure was restricted to a short period so that the impact of nicotine history on SCHZ symptomatology could be evaluated.

**Highlighted version (Discussion, page 28, line 674)**

The association between tobacco smoking and SCHZ has gained attention in recent years [115]. However, scant studies in prodrome patients and in animal models of SCHZ investigated this comorbidity during adolescence, which has the potential to identify mechanisms of nicotine interference in the development of SCHZ.

*2. The authors utilize a statistical approach that requires further explanation. The authors cite the use of both Fisher’s PLSD and relaxed criteria for qualifying interactions. There are several places in the Results section where the interactions are subthreshold (p=0.056, p.13; p=0.083, p.14; p=0.07 and 0.054, p.15), and still probed for significance changes without adjusting for multiple pairwise comparisons. How do the authors’ justify this approach? Planned comparisons, data transformations, outlier detection all seem to be better options for consideration. Careful detail/justification should be included in the Statistics paragraph of the Methods section.*

**Response:** In two-factorial experiments or in more complex factorials, it frequently happens that a limited proportion of the comparisons comprising the main effects have substantial interactions while most of the comparisons have negligible interactions. That being the case, the F-test of the interaction sum of squares as a whole is not an adequate guide as to whether interactions can be disregarded. It has then been suggested that the two-way tables of treatment totals or means should be analyzed before discarding the possibility that no interactions are present, particularly if F is larger than 1 (Snedecor & Cochran, Iowa State Univ. Press, Ames Iowa, 1967 Chapter 12, p.348). We would also like to point out that the criterion for interaction terms was not used to assign significance to the effects but rather to identify interactive factors requiring subdivision for lower-order tests of main effects of *Phencyclidine* and/or *Nicotine*, the factors of chief interest, and that this procedure has been previously used in several studies (e.g. Abreu-Villaça et al., 2008, Neuropsychopharmacology 33(3):599-610; Adigun et al., 2010, Brain Res 1329: 36-44; Adigun et al., 2010. Environ Health Perspect 118: 210-5; Nunes-Freitas et al., 2011. Toxicol Sci 123: 144-54; Ribeiro-Carvalho et al., 2020. PLoS One 15:e0239017; Slotkin et al., 2007. Neuropsychopharmacology 32(5):1082-97). This information was added to the methods section.

**Highlighted version (Methods, page 13, line 355)**

Significance was assumed at the level of p < 0.05 for main effects; however, for interactions at p < 0.1, we also examined whether lower-order main effects were detectable after subdivision of the interactive variables [79]. The criterion for interaction terms was not used to assign significance to the effects but rather to identify interactive factors requiring subdivision for lower-order tests of main effects of Phencyclidine and/or Nicotine, the factors of chief interest [79].

*3. It is not clear whether the study was intended to explore sex differences? There is hardly any rationale given and no hypothesis stated in the Introduction. Moreover, the approach employed for analyzing sex differences appears inconsistent (e.g., why only probe sex differences in novel object recognition?). Thus, it is left unclear why other behavioral analyses in this study collapsed across the sex variable.*

**Response:** Studies from my research group use both sexes. This approach, initially based on an intuitive notion that males behave differently than females, was recently reinforced by NIH recommendation to balance sex in preclinical studies. Over-reliance on male animals and cells might obscure sex differences that could guide clinical studies (Clayton & Collins 2014. Nature 509(7500):282-3. doi: 10.1038/509282a). Besides, previous evidence of sex-differences in the severity of symptoms in SCHZ patients adds to NIH arguments and further justifies the inclusion of both male and female mice. It should be noted, however, that the Reviewer is correct in pointing out that this study was not designed to have sex differences as its main focus, and this is why potential sex-differences were not mentioned in the introduction. Despite that, the Reviewer’s comment seems to indicate that the manuscript would benefit from the addition of a rationale statement that explains the relevance of using both sexes. The text of the methods section was modified accordingly.

**Highlighted version (Methods, page 6, line 175)**

Animals were derived from a C57BL/6 colony maintained at the Universidade Federal Fluminense (Niteroi, Brazil) for over 60 generations. Considering NIH recommendations [49] and previous evidence of sex differences in the severity of symptoms in SCHZ patients [50], both males and females were used.

**Response:** Regarding the Reviewer’s comment on the statistical approach, even though both sexes were used in the study and *Sex* was considered a factor in the statistical analysis, separate analysis for males and females were run only when there were significant interactions between *Sex* and *Phencyclidine* and/or *Nicotine*. For the sake of simplicity, we chose to show data separated by sex only when justified by the statistical analysis. Accordingly, in the novel object recognition test, a significant *Session × Phencyclidine × Nicotine × Sex* interaction was identified and confirmed in the Test session with a lower-order ANOVA (*Phencyclidine* × *Nicotine* × *Sex*). As a result, males and females were analyzed separately and the data were shown in separate figures for males and females. This information was added to the Methods section of the revised version of the manuscript.

**Highlighted version (Methods, page 13, line 349)**

Lower-order uANOVAs, Fisher Protected Least Significant Difference (FPLSD) tests or paired t-tests were used post hoc where appropriate. Figures were segmented by sex specifically when significant Sex interactions were observed.

*4. In my opinion, the Discussion section is too long. There are several interesting points made regarding the neurobiological effects that may be contributing to the behavioral findings, but none of these were specifically examined. The authors may want to consolidate this information for brevity and conciseness. Of course, if data are available to speak to any of these proposed changes, it would greatly enhance the interpretability of the neurobiological pathways they propose.*

**Response:** The discussion section was shortened as suggested.

**Highlighted version (Discussion, page 21, line 519)**

In turn, in rats treated with nicotine during adolescence but not at adulthood, a marked decrease in the expression of accumbal D1 receptors was evident following exposure, which lasted until young adulthood [80]. ~~D1 and D2 receptors are in great part segregated in the nucleus accumbens, however, there is evidence that their~~ Considering that the coordinated activation of D1 and D2 receptors is necessary for proper reward-guided behaviors to take place [81]~~. Accordingly~~, this D1 receptors downregulation may counterbalance the D2 overactivation (…)

**Highlighted version (Discussion, page 23, line 554)**

Glucocorticoids modulation of extracellular dopamine levels in the mesocorticolimbic system has been ~~was~~ demonstrated ~~by the attenuation of stress-evoked increases in dopamine efflux after infusion of a glucocorticoid receptor antagonist into the medial prefrontal cortex~~ [85]~~, and in the nucleus accumbens after adrenalectomy~~ [86] ~~of rats. These findings~~ , which provides a mechanistic explanation for the interference of the hypothalamic–pituitary–adrenal axis in the dopaminergic tone of the mesocorticolimbic system and suggests that stressful situations hamper beneficial effects of nicotine on the positive symptomatology of SCHZ.

**Highlighted version (Discussion, page 24, line 592)**

(…) which might be improved by the modulation of dopaminergic function evoked by drugs of abuse [90,91]. ~~As mentioned before, nAChRs are highly expressed in postsynaptic locations of VTA dopaminergic neurons, and in glutamatergic and GABAergic presynaptic terminals [92-94]. Besides,~~ In this regard, recent evidence points to an important role of both α7 and α4β2 nAChRs in the beneficial effect of acute nicotine in social behavior impairment in adult mice exposed to phencyclidine [78].

**Highlighted version (Discussion, page 25, line 619)**

(…) exhibited downregulation of nAChRs in the frontal cortex, ~~which is in accordance with postmortem evaluations [101,103] and neuroimaging studies [83,85] in SCHZ patients. Interestingly,~~ however, the expected increase (…)

**Highlighted version (Discussion, page 26, line 625)**

~~Considering the involvement of the cholinergic system in cognition processes, including rodents’ NOR [75,104], these results suggest that SCHZ-like cognitive deficits in adolescent mice are not substantially ameliorated by nicotine.~~ Future studies are needed (…)

**Highlighted version (Discussion, page 26, line 638)**

Both α4β2 and α7 nAChRs are involved in the PPI phenomenon [106-108], ~~however, the role of these nAChRs and the brain regions in which their functioning is relevant to PPI and to the nicotine-mediated effects in PPI are yet to be determined [107]. Notwithstanding the gaps in the literature, the involvement of these receptors~~ which suggests that neurobiological changes in the early course of SCHZ are alterable by nicotine.

*5. The authors make the case that the prodromal period of schizophrenia (preceding the onset in adulthood) justifies their approach in employing phencyclidine treatment during the adolescence. It is not clear from the information provided whether this period of susceptibility includes (or is driven by) dysregulation of NMDA-receptor/glutamatergic signaling that would be relevant to their model. This should be described in more detail in the Introduction.*

**Response:** The dysregulation of the glutamatergic system has been suggested to play a central role in SCHZ onset in prodrome/high-risk individuals (Allen et al., 2019. Front Psychiatry 10:298. doi: 10.3389/fpsyt.2019.00298; Parellada & Grassó, 2021. Transl Psychiatry 11(1):271. doi: 10.1038/s41398-021-01385-9; Wenneberg et al., 2020. Schizophr Res 215: 38-48. doi: 10.1016/j.schres.2019.10.050). This information was added to the introduction.

**Highlighted version (Introduction, page 4, line 133)**

(…) Repeated administration of rodents with phencyclidine, a non-competitive NMDA receptor antagonist, is considered the best validated and most widely used model in SCHZ research [40,41]. It produces behavioral alterations associated with the three core (positive, negative and cognitive) symptoms of SCHZ [42]; reduces prepulse inhibition (PPI), a SCHZ endophenotype [43,44] and evokes neurochemical abnormalities associated with SCHZ such as hyperactivity of the mesolimbic dopaminergic pathway and hypoactivity of the mesocortical one [42,45]. Particularly relevant to the current study, the dysregulation of the glutamatergic system has been suggested to play a role in the progression of SCHZ from the prodromal phase to psychosis onset [46–48]

*5’. More generally, caveats regarding the phencyclidine model of schizophrenia should be mentioned.*

**Response:** Caveats of the phencyclidine model of SCHZ are now discussed.

**Highlighted version (Discussion, page 27, line 662)**

Despite its face, construct and predictive validities [40,42,112,113], the PCP model has caveats that should be taken in consideration. Some SCHZ symptoms are of human nature and, as such, a challenge to be modeled [114]. In contrast to pharmacological models, SCHZ is chronic and episodic, with different symptoms predominating at different stages of the disease [113]. Finally, even though the PCP model evokes adaptation mechanisms that correlate with findings obtained in patients with SCHZ, it relies on a single neurotransmitter construct [114]. Limitations are inherent to animal models and reinforce the need of studying SCHZ using multiple models associated with varied but complimentary characteristics of SCHZ.

*6. How do the authors interpret the reduction in “Center Time” exhibited by saline-saline control animals in the open field study on subsequent testing days? Is it possible that this may reflect an acclimation to the testing environment rather than a gauge of emotional responses in subsequent days of testing? If so, the results should provide a clearer delineation between what data relate to anxiety-like behavior (Day 1) versus habituation processes (Days 2 and 3).*

**Response:** We thank the Reviewer for this comment. The time spent in the center of an open field arena (Time Cn) is classically used as a measure of anxiety-like behavior. However, a basic premise of anxiety measures in animal models is that environmental novelty simultaneously evokes fear and curiosity, which creates a typical approach-avoid conflict (Rodgers & Dalvi, 1997. Neurosci Biobehav Rev 21(6):801-10. doi: 10.1016/s0149-7634(96)00058-9). Accordingly, retesting is expected to reduce this conflict and alter both behavioral baselines and the nature of emotional responses to the test apparatus, which has been shown to occur in different models of anxiety (Hascoet et al. 1997. Pharmacol Biochem Behav 58(4):1131-8. doi: 10.1016/s0091-3057(97)00093-2; Holmes et al., 2001. Behav Brain Res 122(2):159-67. doi: 10.1016/s0166-4328(01)00184-x; Holmes & Rodgers. 1998. Pharmacol Biochem Behav 60:473-88. doi: 10.1016/s0091-3057(98)00008-2). These data indicate that the use of Time Cn at days 2 and 3 of the open field test may not be reliable as a measure of anxiety and is consistent with extensive literature that adopts one day testing in the open field (e.g. Abreu-Villaça et al., 2013. Drug Alcohol Depend 133:52–60. doi:10.1016/j.drugalcdep.2013.05.033; Prut & Belzung, 2003. Eur J Pharmacol 463:3–33. doi:10.1016/S0014-2999(03)01272-X; Medlej et al., 2019. Methods Mol Biol 2011:345-367. doi: 10.1007/978-1-4939-9554-7_20). The text of the manuscript was modified to indicate that Time Cn at day 1 was used as a measure of anxiety-like behavior.

**Highlighted version (Methods, page 8, line 229)**

The OF was also used to assess anxiety-like behavior. However, considering evidence that retesting may alter both behavioral baselines and the nature of emotional responses in animal models of anxiety [66–68], anxiety-like behavior was assessed at PN48.

**Highlighted version (Methods, page 13, line 343)**

rANOVAs were performed for the analyses of body mass, OF (Ambulation ~~and Time Cn~~), NOR (PI) as well as PPI (% Inhibition) variables. uANOVAs were performed for Time Cn in the OF and for the TCS variables (%Time, Latency and Entries).

**Highlighted version (Results, page 16, line 394)**

~~The rANOVA demonstrated that phencyclidine treatment and nicotine history interacted affecting the anxiety-like behavior. The analysis also indicated that the impact of this interaction was dependent on the day of the test (Time Cn: Day × Phencyclidine × Nicotine – F~~_~~2,204~~_ ~~= 3.6,~~ *~~p~~* ~~= 0.030). Accordingly, each day was analyzed separately. Lower order~~ Time Cn on day 1, was used do assess anxiety-like behavior. The uANOVA ~~on day 1~~ (Time Cn: Phencyclidine × Nicotine – F_1,102_ = 3.1, *p* = 0.083) identified reductions in the time spent in the center of the OF arena in response to phencyclidine treatment (CT > PCP, *p* = 0.042, FPLSD), nicotine history (CT > NIC, *p* = 0.008, FPLSD) and the combined insult (CT > PCP-NIC, *p* = 0.041, FPLSD) (Fig ~~1~~2B), which indicates anxiogenic effects. ~~Reductions of similar magnitude were identified on day 2, however, the high variability of the data prevented the results from reaching statistical significance. On day 3, there were no significant differences between the experimental groups. Day by day anxiety levels of also varied as a function of the experimental group. In this regard, a significant reduction in Time Cn was identified in the CT group (day 1 to day 3 and day 2 to day 3 –~~ *~~p~~* ~~< 0.0001, paired t-test) but was not evident in the other experimental groups. This unequal day-to-day reduction contributed to the lack of differences between groups on day 3.~~

*7. Given that nicotine reduces some of the behavioral symptoms of phencyclidine-induced schizophrenia, an intriguing possibility is that nicotine may be used as a form of self-medication during this prodromal phase, but runs the risk of creating dependency to something that is harmful in the long-run. Is there evidence that agonists of nicotinic receptors (varencline, nicotine patch) are effective in treating schizophrenic patients?*

**Response:** As novel therapeutic options to SCHZ, recent work has explored α7 nAChRs ligands and positive allosteric modulators, which, in animal models and clinical trials, have a positive impact on cognition and other processes relevant to SCHZ such as PPI and auditory-evoked gating (Terry & Callahan, 2020. Neuropharmacology 170:108053. doi: 10.1016/j.neuropharm.2020.108053; Tregellas & Wylie, 2019. Nicotine Tob Res 21(3):349-356. doi: 10.1093/ntr/nty034). Regarding varenicline, despite the fact that most studies that involve patients with SCHZ investigate its effectiveness as a smoking cessation aid for which, overall, evidence supports its efficacy and safety, there is evidence of a positive impact on affective and cognitive deficits during smoking abstinence (Liu et al., 2011. Psychiatry Res 190: 79–84. doi:10.1016/j.psychres.2011.04.018) and of improved cognitive function when combined with antipsychotic drugs (Hong et al., 2011. Arch Gen Psychiatry 68: 1195–1206. doi:10.1001/archgenpsychiatry.2011.83; Shim et al., 2012. Neuropsychopharmacology 37:660–668). In rats, varenicline effectiveness in treating cognitive impairments in a ketamine-based SCHZ model was shown to be mediated by both α4β2- and α7 nAChRs activation (Potasiewicz et al, 2018. J Psychopharmacol 2018; 33(1):269881118812097. doi: 10.1177/0269881118812097). Unfortunately, there is a lack of studies that investigate varenicline effects on positive and negative symptoms as well as of studies that were carried out during adolescence, both in humans and animal models. The manuscript was modified to include a brief discussion of the possibility of testing these drugs in SCHZ adolescent animal models.

**Highlighted version (Discussion, page 28, line 685)**

Most importantly, the current findings open new possibilities for the investigation of therapeutic approaches to be used during adolescence. In this regard, future studies in adolescents should focus on α7 ligands and allosteric modulators, which have a positive impact on cognition and other processes relevant to SCHZ [117,118]. Varenicline, an approved smoking cessation aid in adults, has low addictive potential when compared to nicotine [119] and its activity as a full agonist of α7 and a partial agonist for the α4β2 nAChR subtypes may also prove to be useful [120–123].

*Minor Concerns:*

*1. At times, the use of nicotine and NIC is inconsistent.*

**Response:** “NIC” refers to the experimental groups exposed to nicotine. “Nicotine” (with a capital N), refers to the statistical factor, while “nicotine” was used in all other situations. We identified one misuse in the methods section. That was fixed.

**Highlighted version (Methods, page 6, line 185)**

(…) to deliver an initial dose rate of 24 mg/Kg/day of ~~NIC~~ nicotine ~~per day~~.

*2. On several occasions, “Latency” is oddly capitalized in the middle of sentences.*

**Response:** We used capital letters when we referred to the name of the variable [e.g Ambulation (line 267), Time Cen (line 271)]. We rewrote the description of Latency in the methods section to make it clear that Latency is the name of the variable. We also corrected its use in the results and discussion sections.

**Highlighted version (Methods, page 11, line 304)**

The percent time on social side [%Time = (social side / (social side + middle chamber + non-social side)) * 100] and the ~~Latency for the~~ time to first entry into the social side (Latency) were used as measures of social interaction. Entries (number of entries in social side + non-social side) was used as a measure of activity.

**Highlighted version (Results, page 19, line 457)**

The ~~Latency for the~~ time to first entry into the social side of the TCS apparatus was affected by interactive effects between phencyclidine and nicotine (Latency: Nicotine – F_1,90_ = 6.5, *p* = 0.013; Phencyclidine x Nicotine – F_1,90_ = 6.1, *p* = 0.015).

**Highlighted version (Discussion, page 24, line 585)**

However, ~~Latency for the~~ the time to first entry into the social side of the TCS was increased in PCP mice, which suggests decreased sociability.

*3. “Sensorymotor” (title on Figure 4) is misspelled.*

**Response:** Corrected.

*4. I would suggest moving 2.3 Materials paragraph closer to the beginning of the Methods section.*

**Response:** This topic was moved as suggested.

*5. I would suggest including information regarding the volume dispensed in systemic injections (e.g., 2.5 mg/kg Flunixin, 10 mg/mL injection for instance).*

**Response:** Information regarding the dispensed volume was added to the manuscript.

**Highlighted version (Methods, page 6, line 187)**

(…) both right after closure and on the following day, mice were given s.c. injections of Flunixin (2.5 mg/Kg, s.c. 2.5 mL/Kg body mass) and Enrofloxacin (2.5 mg/Kg, s.c. 2.5 mL/Kg body mass) for pain management and infection prevention.

**Highlighted version (Methods, page 7, line 203)**

(...) daily injections of phencyclidine, an NMDA receptor antagonist used to model SCHZ, at a dose of 2.5 mg/Kg (s.c., dissolved in saline and administered at a volume of 2.5 mL/Kg body mass) [56,57]. On the last day of phencyclidine treatment (PN53), the dose used was 10 mg/Kg (s.c., 2.5 mL/Kg body mass) [58].

**REVIEWER #2:**

We would like to express our thanks to the Reviewer for his contribution to improve the manuscript. Here, we fully considered his/her comments and criticisms. Please note that in addition to highlighting the changes to the manuscript in the revised version, below, we underlined the text that was either included or revised, and we used the strikeout format to indicate texts that were deleted from the revised version.

*1. (…) This is valuable given the strong comorbidity between schizophrenia and tobacco use. The overall implications of this study are also useful as they highlight the fact that nicotine exposure impacts schizophrenia-like symptoms in a PCP animal model of schizophrenia during adolescence.*

**Response:** We appreciate the Reviewer’s opinion of our study.

*What was the rationale for using the PCP model of schizophrenia versus other models?*

**Response:** SCHZ is a complex mental illness, and its etiology and neurobiological mechanisms are not fully understood. Thus, no animal model completely mimics the characteristics of the disease. In this sense, the most consistent hypothesis for SCHZ etiology is the glutamatergic one. There are genetic data that link glutamatergic changes with SCHZ ontogenesis while dopaminergic alterations would be impacted downstream (McCutcheon et al., 2020. JAMA Psychiatry 77:201-210. doi: 10.1001/jamapsychiatry.2019.3360). Imaging tests of prodrome patients further demonstrate that a glutamatergic dysfunction might act as a pathogenic driver for SCHZ onset (Schobel et al., 2013. Neuron 78:81-93. doi: 10.1016/j.neuron.2013.02.011). Thereby, an advantage of PCP model over other SCHZ models is that it simulates events that may drive or take place during the early manifestations of the disease. Finally, the PCP model has face, construct, and predictive validities (Winship et al.m 2018. Can J Psychiatry 64:5-17. doi:10.1177/0706743718773728; Bubeníková-Valešová et al., 2008. Neurosci Biobehav Rev 32:1014–1023. doi:10.1016/j.neubiorev.2008.03.012; Tizabi, 2007. Neurotox Res 12:233–246. doi:10.1007/BF03033907). In accordance with the Reviewer’s suggestion, we added a detailed rationale for using the PCP model in the introduction of the manuscript.

**Highlighted version (Introduction, page 4, line 131)**

Regarding nicotine, the use of subcutaneous osmotic minipumps allows for a constant dose, a controlled period of administration and has been used as a surrogate for tobacco smoking [38,39]. Repeated administration of rodents with phencyclidine, a non-competitive NMDA receptor antagonist, is considered the best validated and most widely used model in SCHZ research [40,41]. It produces behavioral alterations associated with the three core (positive, negative and cognitive) symptoms of SCHZ [42]; reduces prepulse inhibition (PPI), a SCHZ endophenotype [43,44] and evokes neurochemical abnormalities associated with SCHZ such as hyperactivity of the mesolimbic dopaminergic pathway and hypoactivity of the mesocortical one [42,45]. Particularly relevant to the current study, the dysregulation of the glutamatergic system has been suggested to play a role in the progression of SCHZ from the prodromal phase to psychosis onset [46–48].

*What was the rational for testing mice after the end of nicotine exposure versus during nicotine exposure? Would similar results have been obtained?*

**Response:** This is a relevant issue. The objective of this study was to investigate whether a short period of nicotine exposure during adolescence changes the course of SCHZ. To reach this objective, our experimental design needed to fulfill some conditions: 1) Nicotine exposure needed to be restricted to the adolescent period, 2) The spectrum of SCHZ-like symptoms was to be investigated after the end of nicotine exposure, 3) The impact of nicotine history was to be evaluated by comparing mice exposed to phencyclidine (PCP group) with those with a prior exposure to nicotine (PCP-NIC group). Although we agree with the Reviewer in that exposure to nicotine during the behavioral tests could bring interesting information, the extension of the period of nicotine exposure to include the days of behavioral tests would compromise the interpretation of the data. In this regard, previous data demonstrate that short- versus long-term exposures may result in very distinct behavioral and neurochemical alterations (e.g. Abreu-Villaça et al., 2017. Biochem Pharmacol 144:1-17. doi: 10.1016/j.bcp.2017.06.113; Bombardi et al., 2021. Int J Mol Sci 22(9):4775. doi: 10.3390/ijms22094775; Dean et al., 2020 Sci Rep 10(1):8570. doi: 10.1038/s41598-020-65382-6). We also agree with the Reviewer that further studies aiming to deepen our understanding of the basis of nicotine-phencyclidine interactions are needed. However, it is our understanding that the investigation of concomitant phencyclidine and nicotine exposures, while related to the subject covered in our study, would answer a distinct question, and, therefore, entails a distinct study. The manuscript was modified to make it clearer how the methodological approach was designed to answer our experimental question and to point out that future studies are needed to investigate other aspects of nicotine and SCHZ interactions, particularly during adolescence.

**Highlighted version (Introduction, page 5, line 145)**

Here, we used mice models to elucidate the impact of a short course of adolescent nicotine exposure across the spectrum of SCHZ-like symptoms. Phencyclidine administered on a daily basis guaranteed the development of deficits associated with SCHZ. As for nicotine, exposure was restricted to a short period so that the impact of nicotine history on SCHZ symptomatology could be evaluated.

**Highlighted version (Discussion, page 28, line 675)**

(…) scant studies in prodrome patients and in animal models of SCHZ investigate this comorbidity during adolescence, which has the potential to identify mechanisms of nicotine interference in the development of SCHZ.

*What about the impact of nicotine withdrawal on the magnitude of schizophrenia-like symptoms?*

**Response:** We acknowledge that all behavioral tests were conducted after the end of nicotine exposure, i.e. during withdrawal. However, the term “withdrawal” is frequently used as a synonym of “withdrawal syndrome” to define a set of behavioral alterations (somatic and affective) that occur when exposure to a drug of abuse is interrupted in individuals that are dependent. Since we did not assess dependence levels in our animal model, we were conservative and chose to use the terms “prior nicotine exposure” and “nicotine history” instead of the term “withdrawal”. In addition, in mice, while affective symptoms have a quick onset and can be long-lasting, acute somatic withdrawal signs peak 24 h and are no longer evident 48 h after the end of exposure (Perez et al., 2015. Neuropsychopharmacology 40:2327-36. doi: 10.1038/npp.2015.80; Kotagale et al., 2015. Eur J Pharmacol 754:190-8. doi: 10.1016/j.ejphar.2015.02.033). Accordingly, to avoid the possibility of the interference of these signs in the results, a minimum of 4 days of interval between the end of exposure and the behavioral tests was used. This is now more clearly stated in the methods section.

**Highlighted version (Methods, page 8, line 217)**

The behavioral tests were conducted from PN48 to PN53, i.e., after the end of nicotine exposure, but still during the period of phencyclidine treatment. The 4-day interval between the end of nicotine exposure and the beginning of behavioral tests aimed to assess lasting effects of nicotine without the presence of confounding factors associated with acute withdrawal signs, which peak 24 h and are no longer evident 48 h after the end of exposure in mice [60–64].

*Latency to enter the social side was used as measures of social interaction. Typically, a more direct measure of social interaction such as time in the in the interaction zone is used to look a sociability. The lack of an effect in the percent time in the social side does not provide strong support for the conclusion that there was an effect of PCP on sociability.*

**Response:** Agreed. In the revised version, we emphasized that there were no significant differences in the variable %Time and scaled back out conclusions.

**Highlighted version, Discussion, page 24, line 583)**

In the current study, we failed to identify significant differences for the variable %Time, which is classically used to quantify social interaction in the TCS test. However, ~~Latency for the~~ the time to first entry into the social side of the TCS was increased in PCP mice, which suggests decreased sociability.

*Previous work (https://doi.org/10.3389/fpsyt.2013.00038) has shown that adolescent rats require higher nicotine volume to produce similar cotinine levels as adults. Was this considered when selecting the dose of nicotine and method of delivery?*

**Response:** Rodents’ metabolism is faster than humans’. The dose of nicotine was chosen based on a previous study that used minipumps to expose mice to this same dose of nicotine. In that study, treatment with 24 mg/Kg/day produced plasma nicotine levels (Hambsch et al Br J Pharmacol 2014;171(7):1758-71. doi: 10.1111/bph.12578), within the range found in human smokers (Massadeh et al. J Chromatogr Sci 2009;47(2):170-7. doi: 10.1093/chromsci/47.2.170). This information was included in the methods section. We also added the argument suggested by the reviewer, that adolescent rodents require higher nicotine doses to produce similar cotinine levels when compared to adults.

**Highlighted version (Methods, page 7, line 192)**

The dose rate used in the current study was chosen based on a previous study that, by exposing mice to the same dose, produced nicotine serum levels [39] that are within the range of those found in smokers [52,53]. The dose choice also took into consideration evidence that adolescent rodents require higher nicotine doses to produce similar cotinine levels when compared to adults [54,55].

*The authors should clarify if the tests were counterbalanced or the rational for the order in which mice were tested.*

**Response:** The tests were not counterbalanced. The order of the behavioral tests was based on the stress levels mice were submitted during each test. The OF is considered a test that causes little stress. Previous studies from our lab have demonstrated that the OF does not change corticosterone basal levels (Araujo et al., 2020. Toxicol Res 37:115-124. doi: 10.1007/s43188-020-00045-2.). Protocols for the NOR test include a few days of habituation to the test apparatus. The NOR test followed the 3 days of OF because the same arena was used to both behavioral tests, so that the periods of OF testing were used as habituation to the NOR. As for the PPI, it was the last test due to its stressful nature (the animals are repeatedly submitted to sudden, intense auditory stimuli that evoke a reflexive contraction of the skeletal muscles and, for a reliable measure of the startle responses, the animals are restrained for the whole duration of the test) and to its stability (it does not exhibit habituation or extinction over multiple trials being considered a stable SCHZ marker) (Braff, 2010. Curr Top Behav Neurosci; 4: 349-71. doi: 10.1007/7854_2010_61). This information was included in the revised version.

**Highlighted version (Methods, page 8, line 225)**

All mice were submitted to the four behavioral tests described below (Fig 1) (…). The order of the behavioral tests was based on the stress levels mice are submitted during each test. The OF is considered a test that causes little stress [69]. The NOR test followed the OF because the same arena was used to both behavioral tests, and the periods of OF testing were used as habituation to the NOR. The PPI was the last due to its stressful nature (the animals are repeatedly submitted to sudden, intense auditory stimuli and restrained for the whole duration of the test) and to its stability (it does not exhibit habituation or extinction over multiple trials) [44].

___________________________________________________________________________

**REVIEWER #3:**

We would like to express our thanks to the Reviewer for his contribution to improve the manuscript. Here, we fully considered his/her comments and criticisms. Please note that in addition to highlighting the changes to the manuscript in the revised version, below, we underlined the text that was either included or revised, and we used the strikeout format to indicate texts that were deleted from the revised version.

*The topic of the manuscript is highly significant, given the heightened tobacco use during adolescence in individuals with mental health disorders such schizophrenia. There is also a need for understanding of the nicotine mechanisms underlying these effects. Although the topic of study merits importance, the current form of the manuscript requires revision.*

**Response:** We appreciate the Reviewer’s opinion of our study. The manuscript was revised in accordance with the Reviewer’s criticisms.

*Major concerns:*

*1. The timeline of the experiments is thoughtfully planned. However, the timeline of the experiments requires some clarity, specifically regarding the occurrence of injections, overlapping administration of nicotine pump, and behavioral tests. The manuscript would benefit with a summary timeline of testing order and the post-natal dates.*

**Response:** A timeline was added as Figure 1.

*2. The overall experimental design of this study requires clarity. In some data, only males are used and some data both males and females are reported. Were some tests merged with both male and female data? Or were these data separate cohorts of animals?*

**Response:** We thank the reviewer for this comment. Based on his/her comment, we noticed that, in the original version of the manuscript, we failed to mention that both males and females were used throughout the study. Studies from my research group use both sexes. This approach, initially based on an intuitive notion that males behave differently than females in many situations, was recently reinforced by NIH recommendation to balance sex in preclinical studies. Besides, previous evidence of sex-differences in the severity of symptoms in SCHZ patients adds to NIH arguments and further justifies the inclusion of both male and female mice.

Regarding the Reviewer’s comment on the statistical approach, even though both sexes were used in the study and *Sex* was considered a factor in the statistical analysis, separate analysis for males and females were run only when there were significant interactions between *Sex* and *Phencyclidine* and/or *Nicotine*. For the sake of simplicity, we chose to show data separated by sex only when justified by the statistical analysis. Accordingly, in the novel object recognition test, a significant *Session × Phencyclidine × Nicotine × Sex* interaction was identified and confirmed in the Test session with a lower-order ANOVA (*Phencyclidine* × *Nicotine* × *Sex*). As a result, males and females were analyzed separately and the data were shown in separate figures for males and females. This information was added to the methods section of the revised version.

**Highlighted version (Methods, page 6, line 175)**

Animals were derived from a C57BL/6 colony maintained at the Universidade Federal Fluminense (Niteroi, Brazil) for over 60 generations. Considering NIH recommendations [49] and previous evidence of sex differences in the severity of symptoms in SCHZ patients [50], both males and females were used.

**Highlighted version (Methods, page 13, line 349)**

Lower-order uANOVAs, Fisher Protected Least Significant Difference (FPLSD) tests or paired t-tests were used post hoc where appropriate. Figures were segmented by sex specifically when significant Sex interactions were observed.

*3. Throughout the results, the statistical approach used in this study is inconsistent and requires clarity. In some instances (e.g. locomotor activity), several ANOVAs were tested inconsistent with the design approach of the study, increasing the likelihood of false positives. Where proper correction factors applied to multiple comparison tests? Also, the manuscript set the p-value threshold to p=0.1 for an interaction to occur and is unclear if this threshold is correct.*

**Response:** In two-factorial experiments or in more complex factorials, it frequently happens that a limited proportion of the comparisons comprising the main effects have substantial interactions while most of the comparisons have negligible interactions. That being the case, the F-test of the interaction sum of squares as a whole is not an adequate guide as to whether interactions can be disregarded. It has then been suggested that the two-way tables of treatment totals or means should be analyzed before discarding the possibility that no interactions are present, particularly if F is larger than 1 (Snedecor & Cochran, Iowa State Univ. Press, Ames Iowa, 1967 Chapter 12, p.348). We would also like to point out that the criterion for interaction terms was not used to assign significance to the effects but rather to identify interactive factors requiring subdivision for lower-order tests of main effects of *Phencyclidine* and/or *Nicotine*, the factors of chief interest, and that this procedure has been previously used in several studies (e.g. Abreu-Villaça et al., 2008. Neuropsychopharmacology 33(3):599-610; Adigun et al., 2010. Brain Res 1329:36-44; Adigun et al., Environ Health Perspect 2010. 118(2):210-5; Nunes-Freitas et al., 2011. Toxicol Sci 2011, 123(1):144-54; Ribeiro-Carvalho et al., 2020. PLoS One 15(10):e0239017; Slotkin et al., 2007. Neuropsychopharmacology 32(5):1082-97). This information was added to the Methods section.

**Highlighted version (Methods, page 13, line 355)**

Significance was assumed at the level of p < 0.05 for main effects; however, for interactions at p < 0.1, we also examined whether lower-order main effects were detectable after subdivision of the interactive variables [79]. The criterion for interaction terms was not used to assign significance to the effects but rather to identify interactive factors requiring subdivision for lower-order tests of main effects of Phencyclidine and/or Nicotine, the factors of chief interest [79].

*4. Throughout the discussion, much focus is given to systems and circuits of schizophrenia, however, the results of the study do not imply synaptic signaling or changes in neurotransmission. The discussion requires more information regarding similar behavioral findings.*

**Response:** We were careful when discussing the behavioral data due to the fact that there are few studies that used a behavioral design comparable to the one used in the current study, i.e. a short-term nicotine exposure restricted to the adolescent period and the spectrum of SCHZ-like symptoms investigated after its end. However, the Reviewer’s comment seems to indicate that the manuscript would benefit from a change in focus. We made punctual changes in the discussion section aiming to reduce the focus on systems and circuits of SCHZ and increase the focus on behavioral outcomes.

**Highlighted version (Discussion, page 21, line 519)**

In turn, in rats treated with nicotine during adolescence but not at adulthood, a marked decrease in the expression of accumbal D1 receptors was evident following exposure, which lasted until young adulthood [80]. ~~D1 and D2 receptors are in great part segregated in the nucleus accumbens, however, there is evidence that their~~ Considering that the coordinated activation of D1 and D2 receptors is necessary for proper reward-guided behaviors to take place [81]~~. Accordingly~~, this D1 receptors downregulation may counterbalance the D2 overactivation (…)

**Highlighted version (Discussion, page 23, line 554)**

Glucocorticoids modulation of extracellular dopamine levels in the mesocorticolimbic system has been ~~was~~ demonstrated ~~by the attenuation of stress-evoked increases in dopamine efflux after infusion of a glucocorticoid receptor antagonist into the medial prefrontal cortex~~ [85]~~, and in the nucleus accumbens after adrenalectomy~~ [86] ~~of rats. These findings~~ , which provides a mechanistic explanation for the interference of the hypothalamic–pituitary–adrenal axis in the dopaminergic tone of the mesocorticolimbic system and suggests that stressful situations hamper beneficial effects of nicotine on the positive symptomatology of SCHZ.

**Highlighted version (Discussion, page 24, line 592)**

(…) which might be improved by the modulation of dopaminergic function evoked by drugs of abuse [90,91]. ~~As mentioned before, nAChRs are highly expressed in postsynaptic locations of VTA dopaminergic neurons, and in glutamatergic and GABAergic presynaptic terminals [92-94]. Besides,~~ In this regard, recent evidence points to an important role of both α7 and α4β2 nAChRs in the beneficial effect of acute nicotine in social behavior impairment in adult mice exposed to phencyclidine [78].

**Highlighted version (Discussion, page 25, 604)**

This finding is in accordance with previous evidence of more severe cognitive impairment in male SCHZ patients when compared to female ones [50] and with results derived from both neurodevelopmental [97,98] and genetic animal models of SCHZ [97] tested at young adulthood.

**Highlighted version (Discussion, page 25, line 619)**

(…) exhibited downregulation of nAChRs in the frontal cortex, ~~which is in accordance with postmortem evaluations [101,103] and neuroimaging studies [83,85] in SCHZ patients. Interestingly,~~ ; however, the expected increase (…)

**Highlighted version (Discussion, page 26, line 625)**

~~Considering the involvement of the cholinergic system in cognition processes, including rodents’ NOR [75,104], these results suggest that SCHZ-like cognitive deficits in adolescent mice are not substantially ameliorated by nicotine.~~ Future studies are needed (…)

**Highlighted version (Discussion, page 26, line 638)**

Both α4β2 and α7 nAChRs are involved in the PPI phenomenon [106-108], ~~however, the role of these nAChRs and the brain regions in which their functioning is relevant to PPI and to the nicotine-mediated effects in PPI are yet to be determined [107]. Notwithstanding the gaps in the literature, the involvement of these receptors~~ which suggests that neurobiological changes in the early course of SCHZ are alterable by nicotine.

*5. A major limitation of the manuscript is the lack of metabolism assessment of nicotine during adolescence. It is possible that the phencyclidine altered pharmacokinetics during adolescence that may have impacted the findings. The authors may wish to consider this possibility on their discussion.*

Response: We agree with the Reviewer that pharmacokinetics interactions are an important concern. Nicotine metabolism in humans is mainly mediated by CYP2A6. In mice, CYP2A5, which is analogous structural and functionally to the human CYP2A6, is the main pathway of nicotine biotransformation (Meyer & Maurer, 2011. Pharmacogenomics 12:215-33. doi: 10.2217/pgs.10.171; Murphy et al., 2005. Drug Metab Dispos 33(8):1166-73. doi: 10.1124/dmd.105.004549.). Regarding phencyclidine metabolism, in humans, it is mostly associated to CYP3A and CYP1A (Laurenzana & Owens, 1997. Drug Metab Dispos 25(5):557-63; Meyer & Maurer, 2011. Pharmacogenomics 12:215-33. doi: 10.2217/pgs.10.171). In addition, phencyclidine ability to inactivate CYP2B6 has been extensive described (Shebley et al., 2009. Drug Metab Dispos 37:745-52. doi: 10.1124/dmd.108.024661). In mice, repeated exposures decrease its half-life and lower its levels in the brain, pointing out to alterations in biodispositional parameters. Phencyclidine tolerant mice show increased activity of hepatic cytochrome P450 system (Freeman et al., 1984. Pharmacol Biochem Behav 20:373-7. doi: 10.1016/0091-3057(84)90273-9; Nabeshima et al., 1982. J Pharmacol Methods 7:239-53. doi: 10.1016/0160-5402(82)90040-7), however, we failed to find information regarding which P450 family plays a role on phencyclidine metabolism. We also failed to identify studies that investigated pharmacokinetic interactions between phencyclidine and nicotine. This information and the possibility of pharmacokinetic interactions between these drugs are now discussed in the manuscript.

**Highlighted version (Discussion, page 27, line 670)**

Another limitation of the current study is that nicotine and phencyclidine serum levels were not assessed. Accordingly, despite the lack of evidence in the literature, we cannot rule out that pharmacokinetic interactions between these drugs have impacted our results.

*Minor Concerns:*

*1. Please italicize the Latin phrase “ad libitum” in the methods section.*

**Response:** Done.

*2. Please expand the diffusion rate of the nicotine pump in the methods sections.*

**Response:** The information was added to the methods section.

**Highlighted version (Methods, page 6, line 182)**

(…) and an incision was made to permit s.c. insertion of an osmotic minipump (model 1007d, diffusion rate: 0.5 μL/h, Alzet, Cupertino, CA, U.S.A.).

*3. What is the rationale of testing this dose of nicotine?*

**Response:** Rodents’ metabolism is faster than humans’. The dose of nicotine was chosen based on a previous study that used minipumps to expose mice to this same dose of nicotine. In that study, treatment with 24 mg/Kg/day produced plasma nicotine levels (Hambsch et al Br J Pharmacol 2014. 171(7):1758-71. doi: 10.1111/bph.12578), within the range found in human smokers (Massadeh et al. J Chromatogr Sci 2009. 47(2):170-7. doi: 10.1093/chromsci/47.2.170). This information was included in the methods section. We also included an argument suggested by Reviewer 2, that adolescent rodents require higher nicotine doses to produce similar cotinine levels when compared to adults.

**Highlighted version (Methods, page 7, line 192)**

The dose rate used in the current study was chosen based on a previous study that, by exposing mice to the same dose, produced nicotine serum levels [39] that are within the range of those found in smokers [52,53]. The dose choice also took into consideration evidence that adolescent rodents require higher nicotine doses to produce similar cotinine levels when compared to adults [54,55]

*3’. Is the dose salt form or free base?*

**Response:** Nicotine *free base* was used.

**Highlighted version (Methods, page 6, line 164)**

Nicotine free base was purchased from Sigma Chemical Co. (St. Louis, MO, USA).

**Highlighted version (Methods, page 6, line 184)**

Minipumps were prepared on the day preceding implantation with nicotine free base diluted in milli-Q water (…)

*4. In the methods, how was flunixin and Enrofloxacino administered?*

**Response:** Both flunixin and enrofloxacin were administered subcutaneously. This information was included in the revised version of the manuscript.

**Highlighted version (Methods, page 6, line 187)**

(…) mice were given s.c. injections of flunixin (2.5 mg/Kg, s.c. 2.5 mL/Kg body mass) and enrofloxacin (2.5 mg/Kg, s.c. 2.5 mL/Kg body mass) for pain management and infection prevention.

*5. Please include more detailed information of buffers used to dissolve nicotine and phencyclidine in the methods sections.*

**Response:** Phencyclidine was dissolved in saline. Nicotine was dissolved in deionized water and pH was adjusted to 6.0 with 5M NaOH. This information was added to methods section.

**Highlighted version (Methods, page 6, line 185)**

(…) diluted in milli-Q water (pH adjusted to 6.0 with 5M NaOH).

**Highlighted version (Methods, page 7, line 203)**

(...) daily injections of phencyclidine, an NMDA receptor antagonist used to model SCHZ, at a dose of 2.5 mg/Kg (s.c., dissolved in saline and administered at a volume of 2.5 mL/Kg body mass) [56,57].

*6. Body mass findings would benefit as a table of values.*

**Response:** Body mass means and standard errors are now shown in table 1. Raw data are available as supplementary material.
